# Metagenome analyses identify human endogenous retrovirus–K113 (HML-2) subtype in glioblastoma. Reply.

**DOI:** 10.1172/JCI176406

**Published:** 2023-12-15

**Authors:** Vaidya Govindarajan, Jay Chandar, Avindra Nath, Ashish H. Shah

**Affiliations:** 1 Miller School of Medicine, Department of Neurological Surgery, University of Miami, Coral Gables, Florida, USA.; 2 National Institute of Neurological Disorders and Stroke, NIH, Bethesda, Maryland, USA.

**Keywords:** Stem cells, Virology, Brain cancer, Neuronal stem cells

## The authors reply:

We read with great interest the letter by Macamo et al. (1), supporting our recent work demonstrating the role of human endogenous retrovirus K (HERV-K) (HML-2) in glioblastoma multiforme (GBM) (2). Using a metagenomics approach, they were able to validate that HERV-K (HML-2) is enriched in GBM and not expressed in normal brain samples. Consistent with our data, the authors suggest that HML-2 is capable of producing retroviral proteins, including HERV-K reverse transcriptase (1). Similarly, we have discovered extracellular reverse transcriptase in neurospheres and cerebrospinal fluid derived from patients with GBM. Although the direct oncologic role of these viral proteins in GBM has yet to be uncovered, our data demonstrated that HML-2 preserves a stem cell niche in GBM.

HML-2 may also have a pleiotropic role in GBM pathogenesis and progression of disease. For example, worse outcomes in patients with elevated HML-2 expression may reflect not only preserved stemness, but also neurotoxic or immunosuppressive influences in the tumor microenvironment. In other clinical models of neurodegenerative disease, such as ALS, HML-2 has been found to contribute to neurotoxicity by activating mTOR (3). Similarly, HML-2 may contribute to the neuronal destruction in the GBM tumor microenvironment through conserved intercellular pathways. Finally, the preserved stemness in HML-2–enriched GBM may also activate an immunosuppressive phenotype in cancer stem cells (CSCs). For example, multiple groups have demonstrated that CSCs silence antitumor immune responses by secreting immunosuppressive cytokines and recruiting myeloid-derived suppressor cells and regulatory T cells (4–6). In some preliminary studies, HML-2 abrogated T cell proliferation and dendritic cell activation by activating IL-10 expression (7). Given the diverse role of GBM CSC-derived exosomes, a dedicated study of the diverse role of HML-2 may be warranted.

**Figure 1:**
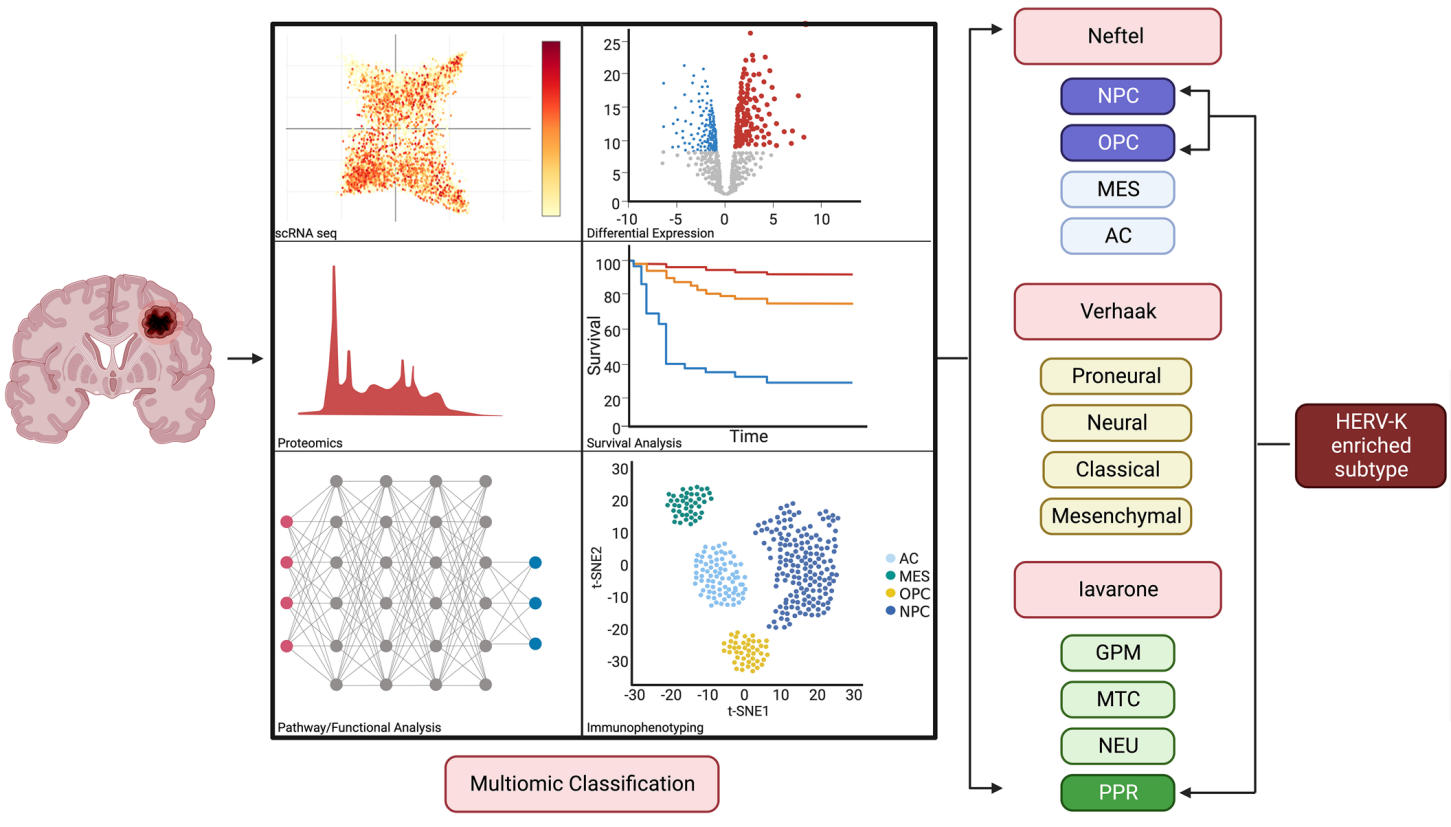
Multiomic classification of GBM based on bulk RNA-Seq, single-cell RNA-Seq, proteomics, and immunophenotyping may confirm novel retroviral-enriched subtype. AC, astrocytes; GPM, glycolytic/plurimetabolic; MES, mesenchymal; MTC, mitochondrial; NEU, neuronal; NPC, neural progenitor cells; OPC, oligodendrocytic precursor cells; PPR, proliferative/progenitor; scRNA-Seq, single-cell RNA-Seq. This figure was created with BioRender.

Finally, based on our results and those from Macamo et al. (1), future studies should focus on investigating the role of HML-2 as a biomarker for clinical outcomes in GBM. Such a study would require a multiomics approach, leveraging HML-2 proteomic, transcriptomic, and epigenetic analyses to cluster patients with GBM by HML-2 expression. HML-2 may define a unique subset of patients with GBM with poor clinical outcomes that may require a tailored therapeutic approach. We propose to redefine GBM classification systems by incorporating HERVs (see Figure 1). In this manner, we may uncover a novel retroviral GBM subtype with distinct clinical and molecular features.

## Version 1 12/15/2023

Electronic publication
